# Supplementary material for: Interlaboratory Concordance of a Multiplex ELISA for Lyme and Lyme-like Illness Using Australian Samples and Commercial Reference Panels: A Proof-of-Concept Study
Source: Pathogens. 2025 Dec 12;14(12):1281. doi: 10.3390/pathogens14121281 (PMC12735738; doi:10.3390/pathogens14121281)
Supplement: Supplementary file 1 [file pathogens-14-01281-s001.zip › pathogens-3967588-supplementary.pdf]

Interlaboratory Concordance of a Multiplex ELISA for Lyme and Lyme-like Illness Using Australian Samples and Commercial Reference Panels: A Proof-of-Concept Study

Kunal Garg[1], Fausto Villavicencio-Aguilar[2], Flora Solano-Rivera[2], and Leona Gilbert[1,4]*

[1]Tezted Ltd, Mattilaniemi 6-8, Jyväskylä, 40100 Finland.

[2]Sanoviv Medical Institute, KM 39 Carretera Libre Tijuana-Ensenada s/n Interior 6, Playas de Rosarito, Baja California, Mexico, 22712.

*** Correspondence:**
Dr Leona Gilbert
leona.gilbert@tezted.com

Supplementary Material

Table S1. Below is a list of serum samples (n=53) indicating whether they were diluted in 30% glycerol (Yes / No). It is important to note that serum samples shared by the Australian lab were not diluted in 30% glycerol. In contrast, the samples shared by Tezted Ltd. were diluted to ensure the preservation of sample quality. Furthermore, the diluted samples in 30% glycerol underwent an additional dilution at a 1:133 ratio in the sample buffer. On the other hand, the undiluted serum samples were diluted at a 1:200 ratio.

**Serum samples diluted in 30% glycerol (Yes / No)**					
AU1	Yes	AU28	Yes	AU52	No
AU2	Yes	AU29	Yes	AU53	No
AU3	Yes	AU30	Yes	AU54	No
AU4	Yes	AU31	No	AU55	No
AU5	Yes	AU32	No	AU56	No
AU6	Yes	AU33	No		
AU10	Yes	AU34	No		
AU11	Yes	AU35	No		
AU12	Yes	AU36	No		
AU13	Yes	AU37	No		
AU14	Yes	AU38	No		
AU15	Yes	AU39	No		
AU16	Yes	AU40	No		
AU17	Yes	AU41	No		
AU18	Yes	AU42	No		
AU19	Yes	AU43	No		
AU20	Yes	AU44	No		
AU21	Yes	AU45	No		
AU22	Yes	AU46	No		
AU23	Yes	AU47	No		

AU24	Yes	AU48	No		
AU25	Yes	AU49	No		
AU26	Yes	AU50	No		
AU27	Yes	AU51	No		

Table S2. All IgM and IgG plate control values passed the plate validity criteria at Tezted Ltd (FIN) and Royal North Shore Hospital (AUS).

Plate #	IgM plate controls (FIN)			IgG plate controls (FIN)			IgM plate controls (AUS)			IgG plate controls (AUS)		
	POS	NEG	R2	POS	NEG	R2	POS	NEG	R2	POS	NEG	R2
Plate 1	4.000	0.047	0.992	3.218	0.048	0.829	4.564	0.039	0.979	2.266	0.042	0.778
Plate 2	4.000	0.049	0.976	3.623	0.052	0.839	4.278	0.040	0.983	1.868	0.048	0.827
Plate 3	4.000	0.048	0.997	3.681	0.049	0.811	4.471	0.037	0.993	2.145	0.039	0.817
Plate 4	4.000	0.055	0.949	3.381	0.059	0.823	4.289	0.042	0.972	1.945	0.048	0.820
Plate 5	4.000	0.133	0.998	2.526	0.235	0.789	4.436	0.130	0.986	1.995	0.039	0.815
Plate 6	4.000	0.058	0.981	3.047	0.053	0.817	4.217	0.038	0.982	2.268	0.039	0.808
Plate 7	4.000	0.047	0.997	3.065	0.053	0.768	4.208	0.037	0.986	2.320	0.040	0.800
Plate 8	4.000	0.196	0.999	4.000	0.201	0.790	4.556	0.037	0.995	2.398	0.040	0.776
Plate 9	4.000	0.047	0.899	3.136	0.047	0.878	4.337	0.040	0.997	2.162	0.088	0.836
Plate 10	4.000	0.057	0.998	2.917	0.049	0.817	4.239	0.041	0.985	2.191	0.041	0.803
Plate 11	4.000	0.065	0.994	3.067	0.060	0.826	4.606	0.037	0.992	1.849	0.040	0.795
Plate 12	4.000	0.059	0.994	2.723	0.060	0.826	4.573	0.038	0.970	1.999	0.042	0.805
Plate 13	4.000	0.054	0.992	3.644	0.241	0.826	4.452	0.047	0.970	2.205	0.042	0.788
Plate 14	4.000	0.065	0.991	3.068	0.050	0.862	4.629	0.036	0.974	2.181	0.038	0.806
Plate 15	4.000	0.048	0.861	3.106	0.116	0.843	4.163	0.037	0.955	2.391	0.038	0.777

Plate 16	4.000	0.109	0.992	2.964	0.066	0.845	4.543	0.036	0.987	2.666	0.042	0.771
Plate 17	4.000	0.053	0.969	2.460	0.177	0.840	N/A	N/A	N/A	N/A	N/A	N/A
Plate 18	4.000	0.058	0.962	2.640	0.056	0.794	N/A	N/A	N/A	N/A	N/A	N/A
Plate 19	4.000	0.061	0.998	3.243	0.067	0.818	N/A	N/A	N/A	N/A	N/A	N/A
Plate 20	4.000	0.049	0.998	2.981	0.052	0.770	N/A	N/A	N/A	N/A	N/A	N/A
Plate 21	4.000	0.045	0.960	2.220	0.086	0.800	N/A	N/A	N/A	N/A	N/A	N/A
Plate 22	4.000	0.056	0.975	2.430	0.057	0.922	N/A	N/A	N/A	N/A	N/A	N/A
Plate 23	4.000	0.061	0.999	2.786	0.078	0.911	N/A	N/A	N/A	N/A	N/A	N/A
**CV%**	**0.000**	**52.844**	**3.509**	**14.434**	**70.937**	**4.667**	**3.647**	**51.742**	**1.130**	**9.873**	**27.262**	**2.413**

N/A = Not Applicable

Table S3. IgM clinical outcomes for specimens (n=28) were performed at Tezted Ltd (FIN) and Royal North Shore Hospital (AUS) for evaluating inter-lab agreement (Figure 1) and clinical assessment between the index (TICKPLEX®) and reference

tests (Figure 2). Table S3 demonstrates the optical density index (ODI) for each repeat and their mean at Tezted Ltd (FIN) and Royal North Shore Hospital (AUS). Positive, negative, and borderline immune responses are highlighted in red, green, and yellow colors.

Specimen ID	*Borrelia afzelii, Borrelia burgdorferi, and Borrelia garinii*		*Borrelia afzelii, Borrelia burgdorferi, and Borrelia garinii* persistent forms		*Babesia microti*		*Bartonella henselae*		*Ehrlichia chaffeensis*		*Rickettsia akari*		Coxsackievirus		Epstein-Barr virus		Human parvovirus B19		*Mycoplasma fermentans* and *Mycoplasma pneumoniae*	
	FIN	AUS	FIN	AUS	FIN	AUS	FIN	AUS	FIN	AUS	FIN	AUS	FIN	AUS	FIN	AUS	FIN	AUS	FIN	AUS
AU3 REPEAT 1	1.076	1.141	1.036	1.124	0.634	0.500	0.608	0.500	0.671	0.743	0.765	0.572	0.928	0.620	0.550	0.336	0.637	0.484	0.649	0.459
AU3 REPEAT 2	1.181	1.049	1.102	0.951	0.694	0.476	0.729	0.486	0.702	0.714	0.784	0.536	5.624	0.628	0.911	0.340	0.843	0.520	0.747	0.510
AU3 MEAN ODI	1.129	1.095	1.069	1.037	0.664	0.488	0.669	0.493	0.686	0.728	0.775	0.554	3.276	0.624	0.731	0.338	0.740	0.502	0.698	0.484
AU4 REPEAT 1	0.800	0.562	0.642	0.430	0.678	0.393	0.650	1.019	0.516	1.123	0.607	0.580	0.403	0.644	0.535	0.439	0.469	0.591	0.488	0.504
AU4 REPEAT 2	0.752	2.373	0.624	0.398	0.548	0.481	0.614	0.531	0.527	0.976	0.626	0.394	0.431	0.240	0.617	0.325	0.579	0.361	0.580	0.399
AU4 MEAN ODI	0.776	1.467	0.633	0.414	0.613	0.437	0.632	0.775	0.521	1.049	0.617	0.487	0.417	0.442	0.576	0.382	0.524	0.476	0.534	0.451
AU5 REPEAT 1	1.038	1.242	0.903	0.577	0.667	0.368	0.632	0.388	0.690	0.742	0.683	0.393	0.462	0.938	0.402	0.363	0.537	0.467	0.603	0.380
AU5 REPEAT 2	1.014	0.909	0.850	0.704	0.591	0.346	0.565	0.385	0.698	0.777	0.715	0.479	0.428	0.688	0.439	0.348	0.632	0.358	0.592	0.409
AU5 MEAN ODI	1.026	1.075	0.877	0.641	0.629	0.357	0.599	0.387	0.694	0.760	0.699	0.436	0.445	0.813	0.420	0.355	0.585	0.412	0.598	0.395
AU6 REPEAT 1	7.814	13.077	4.356	13.926	1.925	16.177	1.860	19.967	2.131	10.513	2.480	21.424	1.176	9.279	1.346	13.968	1.897	18.142	2.023	22.071
AU6 REPEAT 2	12.952	15.678	11.164	16.577	12.840	22.046	14.452	26.182	9.128	15.685	16.642	25.454	7.719	11.789	9.249	12.134	13.044	21.843	15.608	22.006
AU6 MEAN ODI	10.383	14.378	7.760	15.252	7.383	19.112	8.156	23.075	5.629	13.099	9.561	23.439	4.448	10.534	5.298	13.051	7.470	19.993	8.816	22.039
AU13 REPEAT 1	1.233	0.697	0.938	0.681	0.871	0.470	0.972	0.521	0.967	0.066	0.917	0.530	0.632	0.374	0.903	0.763	0.664	0.433	0.983	0.586
AU13 REPEAT 2	1.057	1.027	0.983	0.791	0.731	0.501	0.820	0.522	0.903	0.743	0.791	0.544	0.598	0.427	0.922	0.733	0.732	0.463	0.851	0.619
AU13 MEAN ODI	1.145	0.862	0.961	0.736	0.801	0.486	0.896	0.522	0.935	0.404	0.854	0.537	0.615	0.400	0.913	0.748	0.698	0.448	0.917	0.603
AU14 REPEAT 1	2.433	2.170	2.036	1.658	1.484	1.055	1.525	1.109	1.339	1.420	1.455	1.054	1.142	0.810	2.517	2.203	1.038	0.928	1.305	1.749
AU14 REPEAT 2	2.448	2.185	1.992	1.477	1.285	1.050	1.380	2.022	1.255	1.333	1.455	1.159	1.343	0.873	2.424	2.071	1.154	0.927	1.396	1.048
AU14 MEAN ODI	2.440	2.178	2.014	1.568	1.385	1.052	1.453	1.565	1.297	1.376	1.455	1.107	1.243	0.841	2.470	2.137	1.096	0.928	1.351	1.399
AU16 REPEAT 1	3.671	0.902	0.695	0.425	0.591	0.339	0.632	0.368	0.489	0.401	1.999	0.430	0.456	0.245	0.409	0.488	0.632	0.320	0.667	0.343
AU16 REPEAT 2	0.886	0.898	0.646	0.436	0.559	0.339	0.583	0.359	0.478	0.383	0.620	0.430	0.418	0.241	0.435	0.261	0.564	0.321	0.632	0.380
AU16 MEAN ODI	2.279	0.900	0.671	0.431	0.575	0.339	0.608	0.363	0.484	0.392	1.309	0.430	0.437	0.243	0.422	0.375	0.598	0.321	0.649	0.361
AU18 REPEAT 1	1.829	1.164	1.726	0.876	1.796	0.640	1.325	0.598	3.405	2.157	1.758	0.587	1.198	0.432	1.175	0.424	1.333	0.602	1.408	0.668
AU18 REPEAT 2	2.338	1.720	1.904	0.888	1.844	0.665	1.841	0.572	3.398	2.125	1.550	0.604	1.378	0.481	1.379	0.448	1.844	0.602	2.075	0.775
AU18 MEAN ODI	2.083	1.442	1.815	0.882	1.820	0.652	1.583	0.585	3.402	2.141	1.654	0.596	1.288	0.457	1.277	0.436	1.588	0.602	1.741	0.721
AU19 REPEAT 1	1.110	0.932	0.907	0.788	0.785	1.505	0.839	0.452	0.607	0.595	0.810	0.459	0.758	0.521	0.732	0.362	0.690	0.400	0.632	2.004
AU19 REPEAT 2	1.038	0.968	0.863	0.745	0.688	0.611	0.705	0.497	0.603	0.580	0.753	0.462	0.720	0.566	0.673	0.341	1.006	0.412	1.132	0.490
AU19 MEAN ODI	1.074	0.950	0.885	0.766	0.737	1.058	0.772	0.474	0.605	0.588	0.781	0.460	0.739	0.544	0.703	0.352	0.848	0.406	0.882	1.247
AU20 REPEAT 1	1.943	1.539	1.700	1.436	1.086	0.973	1.246	1.211	1.183	1.619	1.094	0.952	1.384	0.885	0.721	3.779	1.001	0.926	1.230	1.071
AU20 REPEAT 2	1.467	1.530	1.669	1.266	1.038	0.996	1.045	0.923	1.157	1.451	1.063	0.912	1.217	0.924	0.721	3.439	0.843	1.015	0.965	0.630
AU20 MEAN ODI	1.705	1.534	1.684	1.351	1.062	0.985	1.146	1.067	1.170	1.535	1.078	0.932	1.301	0.904	0.721	3.609	0.922	0.970	1.098	0.851
AU21 REPEAT 1	1.157	1.101	1.089	1.190	0.667	0.312	0.735	0.312	0.686	0.559	0.639	0.338	0.459	0.236	0.632	0.308	0.606	0.388	0.684	0.330
AU21 REPEAT 2	1.238	1.185	0.992	1.118	0.721	0.317	0.662	0.340	0.645	0.526	0.639	0.355	0.437	0.246	0.561	0.312	0.606	0.392	0.569	0.321
AU21 MEAN ODI	1.198	1.143	1.040	1.154	0.694	0.314	0.699	0.326	0.666	0.543	0.639	0.346	0.448	0.241	0.597	0.310	0.606	0.390	0.626	0.326
AU28 REPEAT 1	0.619	0.396	0.527	0.340	0.570	0.296	0.596	0.332	0.466	0.371	0.620	0.326	0.418	0.221	0.342	0.193	0.485	0.286	0.511	0.307
AU28 REPEAT 2	0.638	0.445	0.500	0.339	6.243	0.337	0.583	0.354	0.728	0.385	0.620	0.355	0.418	0.236	0.387	0.201	0.543	0.291	0.603	0.317
AU28 MEAN ODI	0.629	0.421	0.514	0.339	3.406	0.317	0.590	0.343	0.597	0.378	0.620	0.341	0.418	0.228	0.364	0.197	0.514	0.288	0.557	0.312
AU29 REPEAT 1	1.986	1.079	1.718	0.927	1.382	0.601	1.234	0.531	1.600	0.919	1.246	0.832	2.664	0.656	2.290	1.881	1.565	1.059	1.339	0.717
AU29 REPEAT 2	1.981	1.534	1.611	1.131	1.532	0.879	1.307	0.978	1.540	1.456	1.208	1.044	1.315	0.731	2.342	2.337	1.733	1.052	1.224	0.756
AU29 MEAN ODI	1.983	1.306	1.664	1.029	1.457	0.740	1.270	0.755	1.570	1.188	1.227	0.938	1.990	0.693	2.316	2.109	1.649	1.055	1.282	0.736
AU30 REPEAT 1	0.933	0.310	0.593	0.275	0.656	0.310	0.553	0.329	0.542	0.387	0.702	0.371	0.308	0.238	0.372	0.193	0.453	0.280	0.563	0.356
AU30 REPEAT 2	0.543	0.340	0.447	0.364	0.581	0.345	0.583	0.431	0.516	0.462	0.715	0.412	0.399	0.238	0.442	0.252	0.506	0.306	0.609	0.322
AU30 MEAN ODI	0.738	0.325	0.520	0.320	0.618	0.327	0.568	0.380	0.529	0.425	0.708	0.391	0.354	0.238	0.407	0.222	0.479	0.293	0.586	0.339
AU31 REPEAT 1	19.048	18.695	17.707	15.609	21.508	20.078	24.310	20.039	15.169	14.024	25.302	23.907	12.582	11.650	14.870	14.327	21.073	20.640	22.987	22.333
AU31 REPEAT 2	19.048	18.830	17.707	15.025	21.508	20.444	24.310	22.616	15.169	14.477	25.302	24.600	12.582	11.552	14.870	13.919	21.073	20.240	22.987	21.594
AU31 MEAN ODI	19.048	18.763	17.707	15.317	21.508	20.261	24.310	21.328	15.169	14.251	25.302	24.254	12.582	11.601	14.870	14.123	21.073	20.440	22.987	21.964
AU32 REPEAT 1	1.729	0.834	1.541	0.640	0.468	0.355	3.385	0.373	0.569	0.507	0.582	0.405	0.406	0.217	0.424	0.251	0.495	0.358	0.684	0.379
AU32 REPEAT 2	1.819	0.832	1.452	0.556	0.468	0.370	0.577	0.395	0.554	0.361	0.601	0.419	0.393	0.229	0.402	0.265	0.553	0.375	0.965	2.054
AU32 MEAN ODI	1.774	0.833	1.496	0.598	0.468	0.363	1.981	0.384	0.561	0.434	0.591	0.412	0.399	0.223	0.413	0.258	0.524	0.366	0.825	1.216
AU33 REPEAT 1	1.533	3.328	1.315	3.964	0.570	0.457	0.869	0.511	0.705	0.426	0.677	0.503	1.695	0.272	0.517	0.715	0.748	0.425	0.603	0.505
AU33 REPEAT 2	1.733	3.675	1.408	4.312	0.613	0.457	0.735	0.587	0.686	0.444	0.784	0.584	2.240	0.311	0.599	0.772	0.685	0.450	0.747	0.588
AU33 MEAN ODI	1.633	3.501	1.361	4.138	0.591	0.457	0.802	0.549	0.696	0.435	0.731	0.543	1.968	0.291	0.558	0.743	0.716	0.438	0.675	0.547
AU34 REPEAT 1	0.929	1.955	0.965	1.833	0.489	0.952	0.535	0.823	0.482	0.730	0.626	0.999	0.434	0.561	0.327	0.582	0.511	1.033	0.477	0.635
AU34 REPEAT 2	1.033	2.075	0.877	1.722	0.446	0.798	0.468	1.118	0.436	0.829	0.519	0.872	0.406	0.696	0.327	0.600	0.479	1.120	0.494	0.884
AU34 MEAN ODI	0.981	2.015	0.921	1.777	0.468	0.875	0.501	0.971	0.459	0.779	0.572	0.935	0.420	0.629	0.327	0.591	0.495	1.076	0.486	0.760
AU35 REPEAT 1	1.205	0.685	1.182	0.651	0.887	0.470	0.924	0.466	1.210	0.682	0.841	0.497	0.607	0.299	0.792	0.387	0.932	0.388	0.874	0.502
AU35 REPEAT 2	1.071	0.698	1.085	0.604	0.645	0.432	0.808	0.472	1.115	0.645	0.829	0.540	0.484	0.322	0.651	0.362	0.632	0.412	0.713	0.472
AU35 MEAN ODI	1.138	0.691	1.133	0.627	0.766	0.451	0.866	0.469	1.162	0.664	0.835	0.518	0.546	0.311	0.721	0.375	0.782	0.400	0.793	0.487
AU36 REPEAT 1	2.162	1.543	1.740	0.971	1.328	0.760	1.556	0.879	1.073	0.639	1.607	0.994	1.192	0.494	1.234	0.496	1.396	0.762	1.511	0.784
AU36 REPEAT 2	1.986	1.521	1.620	0.973	1.231	0.774	2.358	0.817	1.240	0.579	1.613	0.952	1.293	0.499	1.294	0.520	1.470	0.769	1.609	0.856
AU36 MEAN ODI	2.074	1.532	1.680	0.972	1.280	0.767	1.957	0.848	1.157	0.609	1.610	0.973	1.243	0.496	1.264	0.508	1.433	0.765	1.560	0.820
AU37 REPEAT 1	2.076	1.217	1.726	1.031	1.371	0.866	1.434	1.070	1.661	1.358	1.322	0.979	1.384	0.753	1.071	0.713	1.391	0.940	1.385	1.031
AU37 REPEAT 2	1.905	1.469	1.558	3.382	1.317	0.961	1.294	1.007	1.672	1.342	1.335	1.001	1.265	0.649	0.918	0.677	1.259	0.905	1.328	1.013
AU37 MEAN ODI	1.990	1.343	1.642	2.206	1.344	0.914	1.364	1.039	1.667	1.350	1.328	0.990	1.324	0.701	0.994	0.695	1.325	0.922	1.356	1.022
AU38 REPEAT 1	0.981	0.580	0.934	0.606	0.764	0.461	0.760	0.500	0.747	0.578	0.810	0.512	0.812	0.447	0.554	0.342	0.748	0.478	0.908	0.511
AU38 REPEAT 2	0.900	0.680	0.952	0.650	0.753	0.474	0.802	0.546	0.781	2.454	0.784	0.500	0.808	0.448	0.506	0.331	0.790	0.538	0.747	3.198
AU38 MEAN ODI	0.940	0.630	0.943	0.628	0.758	0.467	0.781	0.523	0.764	1.516	0.797	0.506	0.810	0.447	0.530	0.337	0.769	0.508	0.828	1.855
AU40 REPEAT 1	1.024	0.796	1.217	0.591	0.769	0.513	0.796	0.549	0.728	0.518	0.829	0.587	0.651	0.375	0.636	0.458	0.680	0.507	0.885	0.541
AU40 REPEAT 2	1.014	0.788	1.014	0.596	0.801	0.516	0.766	0.479	0.694	0.507	0.721	0.545	0.683	0.437	0.610	0.436	0.543	0.481	0.776	0.541
AU40 MEAN ODI	1.019	0.792	1.116	0.594	0.785	0.514	0.781	0.514	0.711	0.512	0.775	0.566	0.667	0.406	0.623	0.447	0.611	0.494	0.830	0.541

AU42 REPEAT 1	0.586	0.355	0.682	0.331	0.645	0.304	0.662	0.349	0.592	0.292	0.759	0.323	0.500	0.216	0.435	0.226	0.753	0.301	0.672	0.307
AU42 REPEAT 2	0.719	0.410	0.580	0.330	0.511	0.319	0.602	0.323	0.497	0.297	0.614	0.346	0.450	0.246	0.457	0.236	0.579	0.515	0.557	0.314
AU42 MEAN ODI	0.652	0.382	0.631	0.330	0.578	0.312	0.632	0.336	0.544	0.294	0.686	0.334	0.475	0.231	0.446	0.231	0.666	0.408	0.615	0.311
AU43 REPEAT 1	1.871	0.826	1.961	0.868	2.264	0.756	2.127	0.820	1.589	0.658	2.296	0.717	5.181	0.520	1.513	0.490	2.044	0.694	2.230	0.677
AU43 REPEAT 2	1.800	0.945	1.806	0.878	1.618	0.786	1.641	0.844	1.396	0.698	1.733	0.883	1.469	0.461	1.190	0.505	1.860	0.780	1.477	0.760
AU43 MEAN ODI	1.836	0.885	1.884	0.873	1.941	0.771	1.884	0.832	1.492	0.678	2.015	0.800	3.325	0.490	1.351	0.498	1.952	0.737	1.853	0.718
AU50 REPEAT 1	2.138	0.918	3.232	0.695	1.403	0.607	1.313	0.602	1.164	0.695	1.309	0.643	1.035	0.419	0.788	0.390	1.027	0.539	1.075	0.655
AU50 REPEAT 2	2.024	1.050	2.005	0.817	1.237	0.593	1.398	0.670	1.251	0.691	1.208	0.664	1.249	0.441	0.788	0.406	1.296	0.584	1.310	0.665
AU50 MEAN ODI	2.081	0.984	2.618	0.756	1.320	0.600	1.355	0.636	1.208	0.693	1.259	0.653	1.142	0.430	0.788	0.398	1.162	0.562	1.192	0.660
AU52 REPEAT 1	0.967	0.632	8.455	0.655	1.032	0.320	0.626	0.402	0.645	0.377	0.658	0.393	0.453	0.227	0.498	0.313	0.701	0.327	0.672	0.357
AU52 REPEAT 2	0.981	0.695	0.704	0.378	0.618	0.363	0.699	0.364	0.599	0.405	0.854	0.423	0.456	0.226	0.498	0.282	0.622	0.359	0.793	0.506
AU52 MEAN ODI	0.974	0.664	4.580	0.516	0.825	0.342	0.662	0.383	0.622	0.391	0.756	0.408	0.455	0.226	0.498	0.298	0.661	0.343	0.733	0.431
AU53 REPEAT 1	1.386	0.835	1.257	0.671	0.871	0.548	1.149	0.563	1.016	0.521	1.170	0.586	0.774	0.325	1.007	0.514	1.112	0.467	1.006	0.557
AU53 REPEAT 2	1.229	0.831	1.244	0.669	0.850	0.517	1.003	0.648	0.872	0.497	1.031	0.572	0.815	0.362	1.093	0.495	1.001	0.483	0.897	0.517
AU53 MEAN ODI	1.307	0.833	1.251	0.670	0.860	0.533	1.076	0.606	0.944	0.509	1.101	0.579	0.794	0.343	1.050	0.504	1.056	0.475	0.951	0.537

Table S4. IgG clinical outcomes for specimens (n=28) were performed at Tezted Ltd (FIN) and Royal North Shore Hospital (AUS) for evaluating inter-lab agreement (Figure 1) and clinical assessment between the index (TICKPLEX®) and reference tests (Figure 2). Table S4 demonstrates the optical density index (ODI) for each repeat and their mean at Tezted Ltd (FIN) and Royal North Shore Hospital (AUS). Positive, negative, and borderline immune responses are highlighted in red, green, and yellow.

Specimen ID	*Borrelia afzelii, Borrelia burgdorferi, and Borrelia garinii*		*Borrelia afzelii, Borrelia burgdorferi, and Borrelia garinii* persistent forms		*Babesia microti*		*Bartonella henselae*		*Ehrlichia chaffeensis*		*Rickettsia akari*		Coxsackievirus		Epstein-Barr virus		Human parvovirus B19		*Mycoplasma fermentans and Mycoplasma pneumoniae*	
	FIN	AUS	FIN	AUS	FIN	AUS	FIN	AUS	FIN	AUS	FIN	AUS	FIN	AUS	FIN	AUS	FIN	AUS	FIN	AUS
AU3 REPEAT 1	1.277	1.190	1.365	1.522	0.158	0.186	0.197	0.208	0.168	0.167	0.180	0.160	0.159	0.124	0.200	0.141	0.143	0.118	0.182	0.151
AU3 REPEAT 2	1.410	1.346	1.685	1.480	0.179	0.191	0.183	0.202	0.150	0.166	0.123	0.167	1.640	0.144	0.170	0.166	0.058	0.137	0.162	0.163
AU3 MEAN ODI	1.344	1.268	1.525	1.501	0.169	0.189	0.190	0.205	0.159	0.167	0.152	0.164	0.900	0.134	0.185	0.154	0.101	0.128	0.172	0.157
AU4 REPEAT 1	0.535	0.720	0.423	0.622	0.221	0.151	0.191	0.156	0.222	0.259	0.168	0.202	0.158	0.167	1.417	1.591	0.131	0.152	0.173	0.209
AU4 REPEAT 2	0.536	0.661	0.483	0.571	0.186	0.222	0.225	0.192	0.150	0.230	0.179	0.174	0.144	0.263	1.250	2.056	0.146	0.108	0.146	0.237
AU4 MEAN ODI	0.536	0.691	0.453	0.597	0.204	0.187	0.208	0.174	0.186	0.245	0.174	0.188	0.151	0.215	1.334	1.824	0.139	0.130	0.160	0.223
AU5 REPEAT 1	1.046	0.944	1.086	1.219	0.257	0.350	0.242	0.296	0.302	0.655	0.225	0.246	0.226	0.224	2.744	6.491	0.203	0.902	0.190	0.397
AU5 REPEAT 2	0.993	1.159	1.278	1.097	0.201	0.263	0.248	0.460	0.294	0.331	0.224	0.162	0.220	0.291	3.908	6.570	0.241	1.030	0.251	0.310
AU5 MEAN ODI	1.020	1.052	1.182	1.158	0.229	0.307	0.245	0.378	0.298	0.493	0.225	0.204	0.223	0.258	3.326	6.531	0.222	0.966	0.221	0.354
AU6 REPEAT 1	0.750	0.244	0.933	0.271	0.190	0.187	0.357	0.214	0.351	0.226	0.425	0.184	0.364	0.216	0.351	0.786	0.330	0.194	0.371	0.275
AU6 REPEAT 2	0.452	0.565	0.369	0.527	0.227	0.385	0.227	0.379	0.256	0.223	0.247	0.302	0.225	0.259	1.034	0.965	0.231	0.173	0.288	0.197
AU6 MEAN ODI	0.547	0.405	0.377	0.527	0.218	0.286	0.285	0.297	0.274	0.225	0.306	0.243	0.263	0.238	0.807	0.876	0.256	0.184	0.294	0.236
AU13 REPEAT 1	0.514	0.331	0.611	0.294	0.211	0.104	0.214	0.124	0.237	0.137	0.192	0.138	0.249	0.097	0.244	0.126	0.205	0.635	0.194	0.148
AU13 REPEAT 2	0.501	0.279	0.581	0.540	0.244	0.163	0.234	0.179	0.248	0.239	0.380	0.138	0.258	0.115	0.235	0.099	0.230	0.084	0.207	0.103
AU13 MEAN ODI	0.508	0.305	0.596	0.417	0.228	0.134	0.224	0.152	0.243	0.188	0.286	0.138	0.254	0.106	0.240	0.113	0.218	0.360	0.201	0.126
AU14 REPEAT 1	0.362	0.422	0.315	0.665	0.212	0.389	0.244	0.291	0.264	0.325	0.213	0.203	0.234	0.295	0.448	0.463	0.238	0.274	0.253	0.266
AU14 REPEAT 2	0.315	0.367	0.305	0.441	0.215	0.256	0.246	0.483	0.259	0.299	0.222	0.206	0.193	0.374	0.301	0.496	0.211	0.198	0.190	0.276
AU14 MEAN ODI	0.339	0.395	0.310	0.553	0.214	0.323	0.245	0.387	0.262	0.312	0.218	0.205	0.214	0.335	0.375	0.480	0.225	0.236	0.222	0.271
AU16 REPEAT 1	0.321	0.610	0.384	0.347	0.250	0.323	0.325	0.266	0.281	0.294	0.298	0.259	0.246	0.243	0.695	0.627	0.251	0.229	0.291	0.237
AU16 REPEAT 2	0.408	0.583	0.381	0.341	0.242	0.457	0.335	0.221	0.251	0.323	0.277	0.297	0.244	0.395	0.688	1.152	0.274	0.265	0.246	0.210
AU16 MEAN ODI	0.365	0.597	0.383	0.344	0.246	0.390	0.330	0.244	0.266	0.309	0.288	0.278	0.245	0.319	0.692	0.890	0.263	0.247	0.269	0.224
AU18 REPEAT 1	1.016	0.492	0.830	0.552	0.969	0.416	0.882	0.501	1.268	0.382	0.659	0.384	1.141	0.437	1.166	0.465	0.645	0.359	0.665	0.382
AU18 REPEAT 2	1.115	0.510	0.778	0.506	0.910	0.444	0.879	0.387	0.928	0.428	0.717	0.367	0.831	0.348	0.786	0.552	0.600	0.373	0.732	0.382
AU18 MEAN ODI	1.066	0.501	0.804	0.529	0.940	0.430	0.881	0.444	1.098	0.405	0.688	0.376	0.986	0.393	0.976	0.509	0.623	0.366	0.699	0.382
AU19 REPEAT 1	1.200	0.547	0.589	0.616	0.343	0.453	0.385	0.333	0.258	0.369	0.283	0.908	0.344	0.371	0.335	0.326	0.435	0.309	0.281	0.403
AU19 REPEAT 2	0.640	0.584	0.569	0.638	0.408	0.445	0.346	0.276	0.485	0.594	0.271	0.311	0.340	0.327	0.378	0.351	0.256	0.283	0.287	0.311
AU19 MEAN ODI	0.920	0.566	0.579	0.627	0.376	0.449	0.366	0.305	0.372	0.482	0.277	0.610	0.342	0.349	0.357	0.339	0.346	0.296	0.284	0.357
AU20 REPEAT 1	0.812	0.336	0.780	0.412	0.293	0.333	0.344	0.395	0.409	0.284	0.320	0.298	0.312	0.269	0.593	0.287	0.301	0.203	0.316	0.327
AU20 REPEAT 2	0.734	0.335	0.628	0.361	0.270	0.301	0.309	0.259	0.365	0.270	0.249	0.294	0.277	0.292	0.548	0.309	0.305	0.224	0.308	0.235
AU20 MEAN ODI	0.773	0.336	0.704	0.387	0.282	0.317	0.327	0.327	0.387	0.277	0.285	0.296	0.295	0.281	0.571	0.298	0.303	0.214	0.312	0.281
AU21 REPEAT 1	0.794	0.663	0.510	0.708	0.198	0.269	0.846	0.186	0.202	0.205	0.199	0.214	0.160	0.207	0.401	0.462	0.157	0.168	0.195	0.217
AU21 REPEAT 2	0.763	0.713	0.657	0.743	0.201	0.245	0.216	0.214	0.231	0.197	0.209	0.200	0.205	0.171	0.394	0.451	0.203	0.160	0.200	0.198
AU21 MEAN ODI	0.779	0.688	0.584	0.726	0.200	0.257	0.531	0.200	0.217	0.201	0.204	0.207	0.183	0.189	0.398	0.457	0.180	0.164	0.198	0.208
AU28 REPEAT 1	0.444	0.769	0.569	0.838	0.136	0.217	0.198	0.333	0.161	0.148	0.197	0.254	0.119	0.186	0.427	1.767	0.134	0.197	0.127	0.248
AU28 REPEAT 2	0.436	0.723	0.530	0.925	0.229	0.223	0.234	0.221	0.134	0.153	0.188	0.240	0.127	0.188	0.452	1.803	0.163	0.319	0.126	0.173
AU28 MEAN ODI	0.440	0.746	0.550	0.882	0.183	0.220	0.216	0.277	0.148	0.151	0.193	0.247	0.123	0.187	0.440	1.785	0.149	0.258	0.127	0.211
AU29 REPEAT 1	0.310	0.156	0.368	0.181	0.940	0.105	0.147	0.096	0.139	0.088	0.152	0.092	0.160	0.080	0.160	0.095	0.156	0.085	0.138	0.093
AU29 REPEAT 2	0.252	0.156	0.278	0.570	0.231	0.147	0.180	0.121	0.235	0.145	0.194	0.100	0.150	0.082	0.198	0.098	0.178	0.080	0.145	0.097
AU29 MEAN ODI	0.281	0.156	0.323	0.376	0.586	0.126	0.164	0.109	0.187	0.117	0.173	0.096	0.155	0.081	0.179	0.097	0.167	0.083	0.142	0.095
AU30 REPEAT 1	2.248	0.168	0.390	0.169	0.307	0.114	0.198	0.122	0.203	0.145	0.231	0.101	0.180	0.383	0.343	0.172	0.262	0.105	0.210	0.120
AU30 REPEAT 2	0.236	0.253	0.222	0.245	0.138	0.146	0.177	0.161	0.304	0.152	0.318	0.135	0.169	0.118	0.327	0.209	0.261	0.108	0.193	0.131
AU30 MEAN ODI	1.242	0.211	0.306	0.207	0.223	0.130	0.188	0.142	0.254	0.149	0.275	0.118	0.175	0.251	0.335	0.191	0.262	0.107	0.202	0.126
AU31 REPEAT 1	0.721	0.435	0.682	0.407	0.302	0.154	0.342	0.178	0.186	0.159	0.385	0.142	0.455	0.120	1.663	1.114	0.195	0.116	0.336	0.148
AU31 REPEAT 2	0.428	0.439	0.728	0.481	0.207	0.219	0.248	0.165	0.164	0.162	0.417	0.168	0.876	0.146	2.313	1.201	0.255	0.123	0.494	0.158
AU31 MEAN ODI	0.690	0.437	0.639	0.444	0.312	0.187	0.325	0.172	0.251	0.161	0.405	0.155	0.588	0.133	2.484	1.158	0.284	0.120	0.400	0.153
AU32 REPEAT 1	1.299	1.384	1.210	1.685	0.170	0.183	0.244	0.178	0.210	0.185	0.241	0.150	0.187	0.131	0.616	0.334	0.146	0.127	0.242	0.160
AU32 REPEAT 2	1.227	1.528	1.052	1.833	0.177	0.183	0.230	0.204	0.579	0.193	0.134	0.175	0.184	0.150	0.587	0.361	0.205	0.135	0.201	0.186
AU32 MEAN ODI	1.263	1.456	1.131	1.759	0.174	0.183	0.237	0.191	0.395	0.189	0.188	0.163	0.186	0.141	0.602	0.348	0.176	0.131	0.222	0.173
AU33 REPEAT 1	0.929	0.271	0.419	0.295	0.204	0.154	0.219	0.151	3.575	0.419	0.196	0.125	0.208	0.118	1.488	0.665	0.193	0.115	0.191	0.129
AU33 REPEAT 2	1.062	0.298	0.166	0.327	0.121	0.161	0.314	0.157	0.167	0.154	0.203	0.130	0.194	0.230	1.706	0.838	0.195	0.125	0.178	0.115
AU33 MEAN ODI	0.996	0.285	0.293	0.311	0.163	0.158	0.267	0.154	1.871	0.287	0.200	0.128	0.201	0.174	1.597	0.752	0.194	0.120	0.185	0.122

AU34 REPEAT 1	0.462	0.741	0.602	0.368	0.317	0.152	0.527	0.143	0.340	0.166	0.122	0.118	0.248	0.101	0.206	0.150	0.273	0.105	0.284	0.116
AU34 REPEAT 2	0.300	0.905	0.331	0.567	0.395	0.145	0.589	0.157	0.331	0.151	0.112	0.180	0.345	0.089	0.277	0.156	0.541	0.102	0.287	0.124
AU34 MEAN ODI	0.381	0.823	0.467	0.468	0.356	0.149	0.558	0.150	0.336	0.159	0.117	0.149	0.297	0.095	0.242	0.153	0.407	0.104	0.286	0.120
AU35 REPEAT 1	1.112	0.698	1.125	2.563	0.189	0.151	0.323	0.155	0.258	0.141	0.227	0.143	0.189	0.110	0.694	0.761	0.170	0.110	0.172	0.135
AU35 REPEAT 2	1.186	0.760	0.995	0.890	0.175	0.146	0.286	0.154	0.170	0.143	0.239	0.136	0.195	0.115	1.079	0.757	0.164	0.109	0.143	0.146
AU35 MEAN ODI	1.149	0.729	1.060	1.727	0.182	0.149	0.305	0.155	0.214	0.142	0.233	0.140	0.192	0.113	0.887	0.759	0.167	0.110	0.158	0.141
AU36 REPEAT 1	0.487	0.528	0.396	0.326	0.183	0.184	0.268	0.213	0.230	0.386	0.118	0.158	0.185	0.138	0.885	0.616	0.193	0.136	0.206	0.154
AU36 REPEAT 2	0.487	0.547	0.350	0.319	0.170	0.176	0.216	0.178	0.223	0.160	0.097	0.153	0.155	0.134	0.630	0.540	0.152	0.129	0.148	0.168
AU36 MEAN ODI	0.487	0.538	0.373	0.323	0.177	0.180	0.242	0.196	0.227	0.273	0.108	0.156	0.170	0.136	0.758	0.578	0.173	0.133	0.177	0.161
AU37 REPEAT 1	0.415	0.451	0.377	0.482	0.193	0.485	0.212	0.479	0.196	0.567	0.238	0.389	0.192	0.443	0.293	0.389	0.146	0.392	0.154	0.429
AU37 REPEAT 2	0.219	0.577	0.680	0.525	0.305	0.508	0.269	0.473	0.290	0.592	0.202	0.375	0.191	0.398	0.372	0.223	0.193	0.359	0.330	0.417
AU37 MEAN ODI	0.317	0.514	0.529	0.504	0.249	0.497	0.241	0.476	0.243	0.580	0.220	0.382	0.192	0.421	0.333	0.306	0.170	0.376	0.242	0.423
AU38 REPEAT 1	0.283	0.519	4.389	0.553	0.257	0.384	0.259	0.324	0.302	0.572	0.288	0.301	0.125	0.364	0.343	0.993	3.746	0.309	0.195	0.334
AU38 REPEAT 2	0.253	0.687	4.587	0.606	0.332	0.380	0.150	0.369	0.318	0.581	0.176	0.300	0.430	0.370	0.640	0.950	0.297	0.275	0.209	0.321
AU38 MEAN ODI	0.268	0.603	4.488	0.580	0.295	0.382	0.205	0.347	0.310	0.577	0.232	0.301	0.278	0.367	0.492	0.972	2.022	0.292	0.202	0.328
AU40 REPEAT 1	0.804	1.302	1.546	1.368	0.326	0.336	0.188	0.293	0.379	0.353	0.122	0.260	0.234	0.268	0.221	0.264	0.209	0.229	0.224	0.281
AU40 REPEAT 2	1.238	1.437	1.644	1.631	0.598	0.316	0.104	0.326	0.244	0.340	0.207	0.280	0.236	0.257	0.220	0.251	0.216	0.256	0.223	0.297
AU40 MEAN ODI	1.021	1.370	1.595	1.500	0.462	0.326	0.146	0.310	0.312	0.347	0.165	0.270	0.235	0.263	0.221	0.258	0.213	0.243	0.224	0.289
AU42 REPEAT 1	0.992	0.741	3.210	0.368	0.174	0.152	0.390	0.143	0.213	0.166	0.192	0.118	0.185	0.101	0.271	0.150	0.159	0.105	0.173	0.116
AU42 REPEAT 2	1.008	0.905	0.736	0.567	0.183	0.145	0.635	0.157	0.216	0.151	0.211	0.180	0.162	0.089	0.241	0.156	0.203	0.102	0.169	0.124
AU42 MEAN ODI	1.000	0.823	1.973	0.468	0.179	0.149	0.513	0.150	0.215	0.159	0.202	0.149	0.174	0.095	0.256	0.153	0.181	0.104	0.171	0.120
AU43 REPEAT 1	0.416	0.416	0.572	0.456	0.212	0.418	5.256	0.399	2.457	0.503	0.186	0.361	0.260	0.356	0.219	0.326	0.248	0.316	1.779	0.382
AU43 REPEAT 2	0.356	0.419	1.053	0.486	0.195	0.469	0.402	0.441	0.592	0.492	0.187	0.350	0.151	0.352	0.263	0.318	0.181	0.306	0.509	0.365
AU43 MEAN ODI	0.386	0.418	0.813	0.471	0.204	0.444	2.829	0.420	1.525	0.498	0.187	0.356	0.206	0.354	0.241	0.322	0.215	0.311	1.144	0.374
AU50 REPEAT 1	0.663	0.474	0.436	0.454	0.390	0.420	0.344	0.386	0.408	0.417	0.437	0.349	0.592	0.383	1.015	0.682	0.474	0.328	0.414	0.377
AU50 REPEAT 2	0.549	0.468	0.404	0.495	0.614	0.401	0.627	0.454	0.641	0.436	0.535	0.347	0.519	0.391	1.280	0.683	0.640	0.312	0.330	0.342
AU50 MEAN ODI	0.606	0.471	0.420	0.475	0.502	0.411	0.486	0.420	0.525	0.427	0.486	0.348	0.556	0.387	1.148	0.683	0.557	0.320	0.372	0.360
AU52 REPEAT 1	0.264	0.218	0.673	0.217	0.279	0.178	0.150	0.184	0.083	0.185	0.157	0.143	0.157	0.158	0.307	0.214	0.255	0.139	0.218	0.157
AU52 REPEAT 2	0.262	0.331	0.503	0.280	0.390	0.170	0.124	0.161	0.127	0.155	0.128	0.136	0.146	0.146	0.422	0.192	0.128	0.132	0.213	0.148
AU52 MEAN ODI	0.263	0.275	0.588	0.249	0.335	0.174	0.137	0.173	0.105	0.170	0.143	0.140	0.152	0.152	0.365	0.203	0.192	0.136	0.216	0.153
AU53 REPEAT 1	0.912	0.402	0.509	0.411	0.202	0.294	0.222	0.280	0.247	0.262	0.209	0.178	0.135	0.231	0.520	0.378	0.206	0.215	0.212	0.259
AU53 REPEAT 2	0.464	0.411	0.470	0.465	0.199	0.285	0.141	0.266	0.270	0.269	0.258	0.238	0.186	0.227	0.506	0.370	0.200	0.222	0.178	0.289
AU53 MEAN ODI	0.688	0.407	0.490	0.438	0.201	0.290	0.182	0.273	0.259	0.266	0.234	0.208	0.161	0.229	0.513	0.374	0.203	0.219	0.195	0.274

Table S5. Reference Lyme borreliosis test results were not derived from individual assays alone but were interpreted for IgM and IgG positivity or negativity according to the CDC two-tier criteria applied by the sample providers (SeraCare and Plasma Services Group). An IgM- or IgG-positive result was classified as overall positive for IgM and IgG combined analysis. Specimen ID AU6 and AU31 are identical specimens with the difference that AU6 was tested after being stored in 30% glycerol, and AU31 was not stored in 30% glycerol at -20°C. As a result, AU31 was not included in the clinical assessment between the index (TICKPLEX®) and reference tests (Figure 2). Additionally, AU37, AU38, AU43, AU50, and AU53 from tables S3 and S4 were not tested for Lyme borreliosis with any reference tests and are not included in the clinical assessment between the index (TICKPLEX®) and reference tests (Figure 2).

Specimen ID	*IgM reference diagnosis*	*IgG reference diagnosis*	Overall reference diagnosis (IgM and IgG combined)
AU3	POS	POS	POS
AU4	NEG	POS	POS
AU5	POS	NEG	POS
AU6	POS	POS	POS
AU13	NEG	NEG	NEG
AU14	POS	NEG	POS
AU16	NEG	NEG	NEG
AU18	POS	NEG	POS
AU19	POS	NEG	POS
AU20	POS	NEG	POS
AU21	POS	POS	POS
AU28	POS	POS	POS
AU29	POS	POS	POS
AU30	NEG	NEG	NEG
AU32	POS	POS	POS
AU33	POS	NEG	POS
AU34	POS	NEG	POS
AU35	POS	POS	POS
AU36	POS	NEG	POS
AU40	NEG	NEG	NEG
AU42	NEG	POS	POS
AU52	NEG	NEG	NEG

Table S6. IgM clinical findings for specimens (n=52) to evaluate the proportion of positive IgM immune responses against all antigens on the index assay (Figure 3) and categorical responses to only Borrelia, Borrelia, and other microbes, and only other microbes (Figure 4). A mean clinical outcome value is presented for common specimens performed at Tezted Ltd (FIN) and Royal North Shore Hospital (AUS) from Table S3.

Specimen ID	*Borrelia afzelii, Borrelia burgdorferi, and Borrelia garinii*	*Borrelia afzelii, Borrelia burgdorferi, and Borrelia garinii persistent forms*	*Babesia microti*	*Bartonella henselae*	*Ehrlichia chaffeensis*	*Rickettsia akari*	*Coxsackievirus*	*Epstein-Barr virus*	*Human parvovirus B19*	*Mycoplasma fermentans and Mycoplasma pneumoniae*
AU1	1.076	1.027	0.828	0.839	0.762	0.854	0.830	0.613	0.795	0.805
AU2	1.262	1.518	1.113	1.252	1.050	1.183	0.645	0.948	0.911	1.161
Mean AU3	1.112	1.053	0.576	0.581	0.707	0.664	1.950	0.534	0.621	0.591
Mean AU4	1.122	0.523	0.525	0.703	0.785	0.552	0.429	0.479	0.500	0.493
Mean AU5	1.051	0.759	0.493	0.493	0.727	0.567	0.629	0.388	0.498	0.496
Mean AU6	12.380	11.506	13.247	15.615	9.364	16.500	7.491	9.174	13.731	15.427
AU10	1.286	2.165	0.903	1.386	0.812	0.999	0.730	0.639	0.811	0.908
AU11	0.914	0.730	0.602	0.693	1.312	0.677	0.701	0.532	0.537	0.649
AU12	2.881	2.528	1.441	1.440	1.077	1.493	1.230	0.989	1.259	1.328
Mean AU13	1.004	0.848	0.643	0.709	0.670	0.695	0.508	0.830	0.573	0.760
Mean AU14	2.309	1.791	1.218	1.509	1.337	1.281	1.042	2.304	1.012	1.375
AU15	1.043	0.943	0.769	0.790	0.849	0.803	0.516	0.613	0.674	0.747
Mean AU16	1.589	0.551	0.457	0.486	0.438	0.870	0.340	0.398	0.459	0.505
AU17	1.690	1.713	1.484	1.434	1.005	1.328	0.887	0.955	1.238	1.299
Mean AU18	1.763	1.348	1.236	1.084	2.771	1.125	0.872	0.856	1.095	1.231
Mean AU19	1.012	0.826	0.897	0.623	0.596	0.621	0.641	0.527	0.627	1.065
Mean AU20	1.620	1.518	1.023	1.106	1.352	1.005	1.103	2.165	0.946	0.974
Mean AU21	1.170	1.097	0.504	0.513	0.604	0.493	0.345	0.453	0.498	0.476
AU22	0.686	0.624	0.387	0.468	0.542	0.727	0.315	0.431	0.395	0.402
AU23	6.410	2.811	16.857	1.197	1.312	0.810	0.739	0.736	0.938	1.598
AU24	2.095	1.912	1.366	1.404	1.058	1.354	0.868	0.896	1.407	1.276
AU25	1.048	0.757	0.613	0.760	0.630	0.626	0.475	0.416	0.622	0.615
AU26	0.986	0.770	0.602	0.918	0.588	0.911	0.488	0.446	0.543	2.770
AU27	1.729	1.470	1.586	1.367	1.505	1.379	0.991	1.033	1.386	1.408
Mean AU28	0.525	0.426	1.862	0.466	0.488	0.480	0.323	0.281	0.401	0.435
Mean AU29	1.645	1.347	1.099	1.012	1.379	1.083	1.341	2.212	1.352	1.009
Mean AU30	0.532	0.420	0.473	0.474	0.477	0.550	0.296	0.315	0.386	0.463
Mean AU32	1.303	1.047	0.415	1.183	0.498	0.502	0.311	0.336	0.445	1.020
Mean AU33	2.567	2.750	0.524	0.676	0.566	0.637	1.129	0.650	0.577	0.611
Mean AU34	1.498	1.349	0.671	0.736	0.619	0.754	0.524	0.459	0.786	0.623
Mean AU35	0.915	0.880	0.609	0.667	0.913	0.677	0.428	0.548	0.591	0.640
Mean AU36	1.803	1.326	1.023	1.403	0.883	1.292	0.869	0.886	1.099	1.190
Mean AU37	1.667	1.924	1.129	1.202	1.508	1.159	1.013	0.845	1.124	1.189
Mean AU38	0.785	0.786	0.613	0.652	1.140	0.652	0.629	0.433	0.639	1.341
AU39	3.505	2.984	2.210	2.510	2.055	1.841	2.321	2.034	2.002	2.017
Mean AU40	0.905	0.855	0.650	0.647	0.612	0.671	0.536	0.535	0.552	0.686
AU41	1.352	1.301	1.124	7.907	0.978	1.132	0.742	0.680	0.969	2.236
Mean AU42	0.517	0.481	0.445	0.484	0.419	0.510	0.353	0.338	0.537	0.463
Mean AU43	1.361	1.378	1.356	1.358	1.085	1.407	1.908	0.924	1.344	1.286
AU44	0.662	0.483	1.436	0.480	0.584	0.829	4.221	1.517	1.580	3.666
AU45	10.371	9.389	11.566	12.319	7.945	12.727	8.361	10.108	13.434	15.591
AU46	4.595	2.382	3.291	1.301	1.456	1.322	1.953	2.706	1.391	1.431
AU47	0.905	0.717	0.543	0.620	0.398	0.601	0.403	0.342	0.500	0.586
AU48	11.324	10.660	10.802	11.523	7.050	9.937	8.292	7.521	11.727	13.200
AU49	2.000	1.660	0.796	0.827	1.896	0.942	1.356	0.721	0.769	1.345
Mean AU50	1.532	1.687	0.960	0.996	0.950	0.956	0.786	0.593	0.862	0.926
AU51	4.005	3.382	2.613	3.124	2.856	3.422	2.608	14.870	2.613	0.287
Mean AU52	0.819	2.548	0.584	0.523	0.506	0.582	0.341	0.398	0.502	0.582
Mean AU53	1.070	0.960	0.696	0.841	0.727	0.840	0.569	0.777	0.765	0.744
AU54	1.357	1.479	1.183	1.143	0.925	1.265	1.331	0.751	1.022	1.276
AU55	1.271	1.372	1.075	0.985	0.679	1.107	1.450	0.673	0.890	1.052
AU56	2.638	2.510	2.645	2.783	1.547	2.568	1.460	1.937	2.091	2.391

Table S7. IgG clinical findings for specimens (n=52) to evaluate the proportion of positive IgG immune responses against all antigens on the index assay (Figure 3) and categorical responses to only Borrelia, Borrelia, and other microbes, and only other microbes (Figure 4). A mean clinical outcome value is presented for common specimens performed at Tezted Ltd (FIN) and Royal North Shore Hospital (AUS) from Table S4.

Specimen ID	*Borrelia afzelii, Borrelia burgdorferi, and Borrelia garinii*	*Borrelia afzelii, Borrelia burgdorferi, and Borrelia garinii persistent forms*	*Babesia microti*	*Bartonella henselae*	*Ehrlichia chaffeensis*	*Rickettsia akari*	*Coxsackievirus*	*Epstein-Barr virus*	*Human parvovirus B19*	*Mycoplasma fermentans and Mycoplasma pneumoniae*
AU1	0.306	0.253	0.158	0.160	0.281	0.176	0.173	0.292	0.133	0.113
AU2	0.445	0.295	0.498	0.239	0.241	0.171	0.207	0.323	0.249	3.120
Mean AU3	1.306	1.513	0.179	0.198	0.163	0.158	0.517	0.169	0.114	0.165
Mean AU4	0.613	0.525	0.195	0.191	0.215	0.181	0.183	1.579	0.134	0.191
Mean AU5	1.036	1.170	0.268	0.312	0.396	0.214	0.240	4.928	0.594	0.287
Mean AU6	0.476	0.452	0.252	0.291	0.249	0.274	0.250	0.841	0.220	0.265
AU10	0.439	0.292	0.213	0.244	0.215	0.210	0.306	0.310	0.203	0.190
AU11	1.115	0.841	0.381	0.443	0.616	0.344	2.431	0.395	0.341	0.350
AU12	1.133	1.401	0.276	0.534	0.319	0.538	2.681	0.581	0.569	0.324
Mean AU13	0.406	0.507	0.181	0.188	0.215	0.212	0.180	0.176	0.289	0.163
Mean AU14	0.367	0.432	0.268	0.316	0.287	0.211	0.274	0.427	0.230	0.246
AU15	0.860	0.828	0.201	0.225	0.256	0.717	0.254	1.064	0.376	0.231
Mean AU16	0.481	0.363	0.318	0.287	0.287	0.283	0.282	0.791	0.255	0.246
AU17	0.382	0.278	0.201	0.180	0.180	0.182	0.162	0.677	0.184	0.131
Mean AU18	0.783	0.667	0.685	0.662	0.752	0.532	0.689	0.742	0.494	0.540
Mean AU19	0.743	0.603	0.412	0.335	0.427	0.443	0.346	0.348	0.321	0.321
Mean AU20	0.554	0.545	0.299	0.327	0.332	0.290	0.288	0.434	0.258	0.297
Mean AU21	0.733	0.655	0.228	0.366	0.209	0.206	0.186	0.427	0.172	0.203
AU22	0.756	0.678	0.302	0.459	0.327	0.473	0.316	0.726	0.403	0.344
AU23	1.433	1.807	0.226	0.216	0.243	0.329	0.359	0.750	0.367	0.199
AU24	0.436	1.411	0.304	0.375	0.344	0.334	0.313	0.734	0.317	0.284
AU25	0.676	0.611	2.737	0.488	0.374	0.428	0.344	1.600	0.374	0.397
AU26	0.403	1.451	0.233	1.867	0.252	1.146	0.357	0.822	0.315	0.393
AU27	0.893	0.875	0.477	0.657	0.540	0.413	0.408	0.872	0.453	0.434
Mean AU28	0.593	0.716	0.201	0.247	0.149	0.220	0.155	1.112	0.203	0.169
Mean AU29	0.219	0.349	0.356	0.136	0.152	0.135	0.118	0.138	0.125	0.118
Mean AU30	0.726	0.257	0.176	0.165	0.201	0.196	0.213	0.263	0.184	0.164
Mean AU32	1.360	1.445	0.178	0.214	0.292	0.175	0.163	0.475	0.153	0.197
Mean AU33	0.640	0.302	0.160	0.210	1.079	0.164	0.188	1.174	0.157	0.153
Mean AU34	0.602	0.467	0.252	0.354	0.247	0.133	0.196	0.197	0.255	0.203
Mean AU35	0.939	1.393	0.165	0.230	0.178	0.186	0.152	0.823	0.138	0.149
Mean AU36	0.512	0.348	0.178	0.219	0.250	0.132	0.153	0.668	0.153	0.169
Mean AU37	0.416	0.516	0.373	0.358	0.411	0.301	0.306	0.319	0.273	0.333
Mean AU38	0.436	2.534	0.338	0.276	0.443	0.266	0.322	0.732	1.157	0.265
AU39	3.082	0.752	0.469	0.418	0.527	0.391	0.354	0.480	0.517	3.147
Mean AU40	1.195	1.547	0.394	0.228	0.329	0.217	0.249	0.239	0.228	0.256
AU41	1.223	1.343	3.627	0.401	0.388	0.262	0.309	0.823	0.248	0.199
Mean AU42	0.912	1.220	0.164	0.331	0.187	0.175	0.134	0.205	0.142	0.146
Mean AU43	0.402	0.642	0.324	1.625	1.011	0.271	0.280	0.282	0.263	0.759
AU44	0.502	4.587	0.326	0.222	0.269	0.339	0.313	0.697	0.509	0.237
AU45	0.726	0.653	0.544	0.598	0.531	0.539	1.211	1.163	0.556	0.467
AU46	0.842	0.712	0.476	1.535	0.292	0.217	0.463	0.722	0.385	0.294
AU47	1.723	1.041	0.405	0.243	0.223	0.275	0.278	1.017	0.212	0.304
AU48	0.499	0.450	0.195	0.997	0.163	0.320	0.387	0.531	0.304	0.500
AU49	0.578	0.619	0.113	0.145	0.071	0.136	0.238	0.134	0.093	0.120
Mean AU50	0.539	0.447	0.456	0.453	0.476	0.417	0.471	0.915	0.439	0.366
AU51	1.402	1.033	4.605	0.714	0.765	0.719	0.581	0.899	0.483	0.546
Mean AU52	0.269	0.418	0.254	0.155	0.138	0.141	0.152	0.284	0.164	0.184
Mean AU53	0.547	0.464	0.245	0.227	0.262	0.221	0.195	0.444	0.211	0.235
AU54	0.368	0.330	0.237	0.210	0.225	0.284	0.156	0.516	0.239	0.223
AU55	0.426	0.353	0.237	0.382	0.252	0.465	0.276	0.513	0.853	0.222
AU56	0.361	0.323	0.230	0.284	0.337	4.860	1.562	3.335	0.350	3.248

Table S8. Clinical correlation of positive IgM responses in Australian sera (n=17) for other microbes in tickborne co-infections and other opportunistic infections to evaluate the proportion of positive IgM immune responses against the RNSH (AUS) reference results. A mean ODIs of Australian specimens performed at Tezted Ltd (FIN) and Royal North Shore Hospital (AUS) from Table S3 were used to compare with the RNSH reference results. The table includes Australian samples (n=17), excluding specimens from Plasma Services Group, USA, and AccuLyme SeraCare panel, France. Positive, negative, and borderline immune responses are highlighted in red, green, and yellow. Agreement refers to whether the index assay result matches the previously established result from Australia; a match is recorded as 'Yes' (Y) and a mismatch as 'No' (N).

RNSH Reference Results	Enterovirus CFT = 64	B.henslae Ab >=2048	B.henslae Ab >=512	B.henslae Ab = 512, B.burgdorferi IgG, IgM Negative	Borrelia IgG Pos, IgM neg	Borrelia IgG Pos, IgM neg	R.australis IFAT >=512	R.australis IFAT >=512, R.typhi >=256	Scrub typhus IFAT >=128	R.australis 128, R.typhi >=256	B.henslae = 256	Scrub typhus >=128	Borrelia IgG Pos, IgM Pos	Parvovirus IgG Pos, IgM Pos, EBNA G Pos, VCA G Pos, VCA M Neg	Parvovirus IgG Pos, IgM Pos, EBNA G Neg, VCA G Neg, VCA M Pos	Neg for all Borrelia, Bartonella, QF, Rickettsia, Leptospira, B.burgdorferi (IgG and IgM negative)	Chlamydia IgA pos , IgG neg		
**IgM**	AU37	AU38	AU39	AU40	AU41	AU42	AU43	AU44	AU45	AU46	AU47	AU48	AU49	AU50	AU51	AU52	AU53	No. of samples in agreement	Total no. compared
Borrelia afzelii, Borrelia burgdorferi, and Borrelia garinii	1,667	0,785	3,505	0,905	1,352	0,517	1,361	0,662	10,371	4,595	0,905	11,324	2,000	1,532	4,005	0,819	1,070		
Agreement?				Y	N	Y							Y			Y		4	5
Borrelia afzelii, Borrelia burgdorferi, and Borrelia garinii persistent forms	1,924	0,786	2,984	0,855	1,301	0,481	1,378	0,483	9,389	2,382	0,717	10,660	1,660	1,687	3,382	2,548	0,960		
Agreement?				Y	N	Y							Y			Y		5	6
Babesia microti	1,129	0,613	2,210	0,650	1,124	0,445	1,356	1,436	11,566	3,291	0,543	10,802	0,796	0,960	2,613	0,584	0,696		
Agreement?																		0	0
Bartonella henselae	1,202	0,652	2,510	0,647	7,907	0,484	1,358	0,480	12,319	1,301	0,620	11,523	0,827	0,996	3,124	0,523	0,841		
Agreement?		N	Y	N							N					Y		2	5
Ehrlichia chaffeensis	1,508	1,140	2,055	0,612	0,978	0,419	1,085	0,584	7,945	1,456	0,398	7,050	1,896	0,950	2,856	0,506	0,727		
Agreement?																		N/A	N/A
Rickettsia akari	1,159	0,652	1,841	0,671	1,132	0,510	1,407	0,829	12,727	1,322	0,601	9,937	0,942	0,956	3,422	0,582	0,840		
Agreement?																		N/A	N/A
Coxsackievirus	1,013	0,629	2,321	0,536	0,742	0,353	1,908	4,221	8,361	1,953	0,403	8,292	1,356	0,786	2,608	0,341	0,569		
Agreement?	Y																	1	1
Epstein-Barr virus	0,845	0,433	2,034	0,535	0,680	0,338	0,924	1,517	10,108	2,706	0,342	7,521	0,721	0,593	14,870	0,398	0,777		
Agreement?														Y	Y			2	2
Human parvovirus B19	1,124	0,639	2,002	0,552	0,969	0,537	1,344	1,580	13,434	1,391	0,500	11,727	0,769	0,862	2,613	0,502	0,765		
Agreement?														N	Y			1	2
Mycoplasma fermentans and Mycoplasma pneumoniae	1,189	1,341	2,017	0,686	2,236	0,463	1,286	3,666	15,591	1,431	0,586	13,200	1,345	0,926	0,287	0,582	0,744		

Table S9. Clinical correlation of positive IgG responses in Australian sera (n=17) for other microbes in tickborne co-infections and other opportunistic infections to evaluate the proportion of positive IgG immune responses against the RNSH (AUS) reference results. A mean ODIs of Australian specimens performed at Tezted Ltd (FIN) and Royal North Shore Hospital (AUS) from Table S4 were compared with the RNSH reference results. The table includes Australian samples (n=17), excluding specimens from Plasma Services Group, USA, and AccuLyme SeraCare panel, France. Positive, negative, and borderline immune responses are highlighted in red, green, and yellow. Agreement refers to whether the index assay result matches the previously established result from Australia; a match is recorded as 'Yes' (Y) and a mismatch as 'No' (N).

RNSH Reference Results	Enterovirus CFT = 64	B.henslae Ab >=2048	B.henslae Ab >=512	B.henslae Ab = 512, B.burgdorferi IgG, IgM Negative	Borrelia IgG Pos, IgM neg	Borrelia IgG Pos, IgM neg	R.australis IFAT >=512	R.australis IFAT >=512, R.typhi >=256	Scrub typhus IFAT >=128	R.australis 128, R.typhi >=256	B.henslae = 256	Scrub typhus >=128	Borrelia IgG Pos, IgM Pos	Parvovirus IgG Pos, IgM Pos, EBNA G Pos, VCA G Pos, VCA M Neg	Parvovirus IgG Pos, IgM Pos, EBNA G Neg, VCA G Neg, VCA M Pos	Neg for all Borrelia, Bartonella, QF, Rickettsia, Leptospira, B.burgdorferi (IgG and IgM negative)	Chlamydia IgA pos , IgG neg		
**IgG**	AU37	AU38	AU39	AU40	AU41	AU42	AU43	AU44	AU45	AU46	AU47	AU48	AU49	AU50	AU51	AU52	AU53	No. of samples in agreement	Total no. compared
Borrelia afzelii, Borrelia burgdorferi, and Borrelia garinii	0,416	0,436	3,082	1,195	1,223	0,912	0,402	0,502	0,726	0,842	1,723	0,499	0,578	0,539	1,402	0,269	0,547		
Agreement?				N	Y	Y							N			Y		3	5
Borrelia afzelii, Borrelia burgdorferi, and Borrelia garinii persistent forms	0,516	2,534	0,752	1,547	1,343	1,220	0,642	4,587	0,653	0,712	1,041	0,450	0,619	0,447	1,033	0,418	0,464		
Agreement?				N	Y	Y							N			Y		3	5
Babesia microti	0,373	0,338	0,469	0,394	3,627	0,164	0,324	0,326	0,544	0,476	0,405	0,195	0,113	0,456	4,605	0,254	0,245		
Agreement?																		0	0
Bartonella henselae	0,358	0,276	0,418	0,228	0,401	0,331	1,625	0,222	0,598	1,535	0,243	0,997	0,145	0,453	0,714	0,155	0,227		
Agreement?		N	N	N							N					Y		1	5
Ehrlichia chaffeensis	0,411	0,443	0,527	0,329	0,388	0,187	1,011	0,269	0,531	0,292	0,223	0,163	0,071	0,476	0,765	0,138	0,262		
Agreement?																		N/A	N/A
Rickettsia akari	0,301	0,266	0,391	0,217	0,262	0,175	0,271	0,339	0,539	0,217	0,275	0,320	0,136	0,417	0,719	0,141	0,221		
*Agreement?																		N/A	N/A
Coxsackievirus	0,306	0,322	0,354	0,249	0,309	0,134	0,280	0,313	1,211	0,463	0,278	0,387	0,238	0,471	0,581	0,152	0,195		
Agreement?	N																	0	1
Epstein-Barr virus	0,319	0,732	0,480	0,239	0,823	0,205	0,282	0,697	1,163	0,722	1,017	0,531	0,134	0,915	0,899	0,284	0,444		
Agreement?														Y	Y			2	2
Human parvovirus B19	0,273	1,157	0,517	0,228	0,248	0,142	0,263	0,509	0,556	0,385	0,212	0,304	0,093	0,439	0,483	0,164	0,211		
Agreement?														N	N			0	2
Mycoplasma fermentans and Mycoplasma pneumoniae	0,333	0,265	3,147	0,256	0,199	0,146	0,759	0,237	0,467	0,294	0,304	0,500	0,120	0,366	0,546	0,184	0,235		
